# Effects of Natural Ingredient Xanthohumol on the Intestinal Microbiota, Metabolic Profiles and Disease Resistance to *Streptococcus agalactiae* in Tilapia *Oreochromis niloticus*

**DOI:** 10.3390/microorganisms13071699

**Published:** 2025-07-20

**Authors:** Aiguo Huang, Yanqin Wei, Jialong Huang, Songlin Luo, Tingyu Wei, Jing Guo, Fali Zhang, Yinghui Wang

**Affiliations:** 1 Guangxi Laboratory on the Study of Coral Reefs in the South China Sea, Coral Reef Research Center of China, School of Marine Sciences, Guangxi University, Nanning 530004, China; aiguo_200891@126.com (A.H.); weiyanqin113@163.com (Y.W.); luosonglin0528@163.com (S.L.);; 2 School of Resources, Environment and Materials, Guangxi University, Nanning 530004, China; 3 Key Laboratory of Environment Change and Resources Use in Beibu Gulf, Ministry of Education, Nanning Normal University, Nanning 530001, China; guojing8925@126.com; 4 Institute of Green and Low Carbon Technology, Guangxi Institute of Industrial Technology, Nanning 530000, China

**Keywords:** *Streptococcus agalactiae*, tilapia, xanthohumol, antibacterial activity, intestinal microbiota, metabolomics

## Abstract

*Streptococcus agalactiae* (SA) is a severe prevalent pathogen, resulting in high morbidity and mortality in the global tilapia industry. With increasing bacterial resistance to antibiotics, alternative strategies are urgently needed. This study aims to investigate the antibacterial activity and the underlying mechanisms of the natural product xanthohumol (XN) against SA infection in tilapia (*Oreochromis niloticus*). The results showed that XN could significantly reduce the bacterial loads of SA in different tissues (liver, spleen and brain) after treatment with different tested concentrations of XN (12.5, 25.0 and 50.0 mg/kg). Moreover, XN could improve the survival rate of SA-infected tilapia. 16S rRNA gene sequencing demonstrated that the alpha-diversity index (Chao1 and Shannon_e) was significantly increased in the XN-treated group (MX group) compared to the SA-infected group (CG group) (*p* < 0.05), and the Simpson diversity index significantly decreased. The Bray–Curtis similarity analysis of non-metric multidimensional scaling (NMDS) and principal coordinate analysis (PCA) showed that there were significant differences in microbial composition among groups. At the phylum level, the relative abundance of the phyla Actinobacteria, Proteobacteria and Bacteroidetes decreased in the MX group compared to the CG group, while the relative abundance of the phyla Fusobacteria, Firmicutes and Verrucomicrobia increased. Differences were also observed at the genus level; the relative abundance of *Mycobacterium* decreased in the MX group, but the abundance of *Cetobacterium* and *Clostridium_sensu_stricto_1* increased. Metabolomics analysis revealed that XN changed the metabolic profile of the liver and significantly enriched aspartate metabolism, glycine and serine metabolism, phosphatidylcholine biosynthesis, arginine and proline metabolism, glutamate metabolism, urea cycle, purine metabolism, methionine metabolism, betaine metabolism, and carnitine synthesis. Correlation analysis indicated an association between the intestinal microbiota and metabolites. In conclusion, XN may be a potential drug for the prevention and treatment of SA infection in tilapia, and its mechanism of action may be related to the regulation of the intestinal microbiota and liver metabolism.

## 1. Introduction

Tilapia is one of the largest fish species globally, and its production is increasing every year. However, the emergence of pathogens has become a major limiting factor for the production and supply of tilapia. Among pathogens, *Streptococcus agalactiae* (SA) is the main bacterial pathogen in tilapia farms. SA infection can result in high mortality rates of up to 30–90% for tilapia, causing severe economic losses for the tilapia aquaculture industry [1]. Antibiotics are used to control SA infection to some extent [2]. However, the abuse of antibiotics leads to the emergence of drug-resistant microbes and antibiotic residues in foods [3]. Thus, it is urgent to identify new prevention and therapy agents against SA infection in tilapia aquaculture.

Medicinal plants and their chemical composition are considered potential candidates for antimicrobial drugs. Many studies have demonstrated that medicinal plants have notable anti-SA activity in tilapia, such as *Aristolochia debilis*, *Panax ginseng*, *Spatholobus suberectus, Scutellaria baicalensis* and *Sophora flavescens* [4,5,6]. Some plant extracts can also regulate the intestinal microbiota and hepatic metabolism of tilapia. Guava and Star gooseberry leaf extracts, whether used alone or in combination, can enhance the intestinal microbial diversity of tilapia infected with *Aeromonas hydrophila*, increase the abundance of probiotics, and combat Aeromonas hydrophila infection [7]. *Bidens pilosa* can regulate the composition of the intestinal microbiota in tilapia, thereby affecting various metabolic pathways in the liver, such as amino acid metabolism, carbohydrate metabolism and lipid metabolism, and promoting growth through the gut–liver axis [8]. Interestingly, the chemical composition of medicinal plants also exhibits antibacterial activity. For instance, baicalin is the main component of *Scutellaria baicalensis*, and it has significant antibacterial activity against SA infection in tilapia by attenuating SA virulence [9]. Xanthohumol (XN), an isoprenylated flavonoid component in *Sophora flavescens*, can regulate inflammation- and apoptosis-related innate immunity and exert multi-directional pro-healing properties on damaged hair cells in zebrafish embryos [10]. Furthermore, XN has a safe concentration in tilapia exceeding 100 mg/kg and can significantly inhibit GBS infection in tilapia [6]. However, previous research only confirmed that XN possesses antibacterial activity, while the underlying mechanisms of its antibacterial activity and its potential associations with intestinal microbiota and hepatic metabolism remain insufficiently studied.

In this study, the antibacterial activity and potential mechanism of XN against SA infection were evaluated in tilapia (GIFT, *Oreochromis niloticus*). The anti-SA activity of XN in tilapia was investigated by quantifying the bacterial loads using quantitative real-time PCR (qPCR) and evaluating the mortality rates of SA-infected tilapia. Furthermore, we employed 16S rRNA sequencing and untargeted metabolomics to identify the changes in intestinal microbiota and liver metabolites, respectively. This study will provide a comprehensive understanding of the regulatory roles of XN in tilapia.

## 2. Materials and Methods

### 2.1. Fish, Bacteria and Chemicals

Tilapia *Oreochromis niloticus* (body weight: 8.85 ± 0.11 g) was purchased from a fish farm in Nanning, Guangxi, China. Fish were acclimatized for 14 days in 200 L aquariums at 30 °C and fed with a commercial pellet diet twice daily. The animal use protocol was approved by the Animal Care and Welfare Committee of Guangxi University (GXU-2023-0531). SA strain GXYL7, originally isolated from tilapia suffering from bacterial disease, was used in our study [11]. Natural compound XN (CAS No. 6754-58-1) was purchased from Aladdin Biochemical Technology Co., Ltd. (Shanghai, China).

### 2.2. SA Quantification

SA quantification was performed using qPCR, as in our previous study [12]. Simply, the *cfb* gene, which encoded the CAMP factor, was selected as the target gene. It was cloned using the primers cfb-F (5′-TAGCTTAGTTATCCCAAATCCC-3′) and cfb-R (5′-TAAAGACTTCATTGCGTGCC-3′) into the pMD19-T simple vector (Takara, Shanghai, China) for the target amplicon. The copy numbers of the target amplicon were quantified by measuring the concentration using a NanoDrop spectrophotometer (NanoDrop Technologies Inc., Wilmington, DE, USA). The target amplicon was diluted and used as the template for qPCR. The qPCR was performed using UltraSYBR Mixture (CWBIO, Taizhou, China) in the ABI 7500 Fast Real-Time PCR System. The PCR cycling conditions were as follows: 95 °C for 10 min, then 40 cycles at 95 °C denaturation for 15 s, followed by annealing at 60 °C for 1 min. To verify the amplification of a single product, a melt curve analysis of 5 s per step from 65 to 95 °C was performed at the end of each PCR thermal profile. Regression of the log of *cfb* gene copy numbers and the corresponding cycle threshold (Ct) value was used as a standard curve to determine the copy numbers of SA.

### 2.3. Experimental Design and Sampling

The anti-SA activity of XN was evaluated in SA-infected tilapia, as in a previous study [6]. The experimental tilapia were grouped using a random number table method and assigned to the control group, infection group, and XN treatment group. We further evaluated the anti-SA activity of XN in tilapia. Tilapia were divided into the control group and XN experimental groups (12.5, 25.0 and 50.0 mg/kg). The control groups were injected with SA only, and the experimental groups were injected with SA suspensions and different concentrations of XN. Each fish was injected with 100 μL suspensions. The final concentration of SA bacterial suspensions was 1 × 10^8^ cfu/mL. The liver, spleen, brain or intestinal tissues were sampled and stored at −80 °C for analysis.

To investigate the preventive and control effects of XN, a 14-day experiment was conducted on tilapia. For the XN-treated groups, tilapia were intraperitoneally injected with a premixed solution of SA and XN, resulting in XN concentrations in tilapia reaching 12.5, 25, and 50 mg/kg (drug weight: fish weight), which were designated as the 12.5 mg/kg XN group (LX), 25 mg/kg XN group (MX), and 50 mg/kg XN group (HX), respectively. The infected group (CG) was injected with SA bacterial solution alone, while the healthy group was injected with PBS solution (CP). Each group was set with 3 replicates, with 15 fish in each replicate. Each fish was injected with 100 μL of the treatment solution, and the final concentration of GBS bacterial solution was 1 × 10^8^ cfu/mL. Mortality was recorded daily.

### 2.4. DNA Extraction and Absolute Quantification of the Copy Numbers of SA Genomic DNA

The 15 mg tissue samples were taken for DNA extraction, and all tissues were triturated using a tissue homogenizer (Sangon Biotech, Shanghai, China). Total genomic DNA was extracted using the TIANamp Bacteria DNA Kit (Tiangen, Sichuan, China) following the manufacturer’s instructions. Extracted DNA was subjected to quantification via NanoDrop spectrophotometer (NanoDrop Technologies Inc., Wilmington, DE, USA). Then, the DNA was used to quantify the copy numbers of SA genomic DNA via qPCR.

### 2.5. 16S rRNA Gene Sequencing for the Intestinal Microbiota Analysis

The genomic DNA of different samples was extracted according to the E.Z.N.A. ^®^Stool DNA Kit (Omega, Inc., Dewitt, NY, USA). PCR amplification was performed in a 25 μL reaction mixture to obtain the V3–V4 regions of 16S rRNA gene using the primers 338F (5′-ACTCCTACGGGAGGCAGCA-3′) and 806R (5′-GGACTACHVGGGTWTCTAAT-3′). The 5′ ends of the primers were tagged with specific barcodes for each sample and were sequenced with universal primers. The PCR condition consisted of an initial denaturation at 94 °C for 5 min; 30 cycles of denaturation at 94 °C for 30 s, annealing at 52 °C for 30 s, and extension at 72 °C for 30 s; and then final extension at 72 °C for 10 min. The PCR product was confirmed using 1% agarose gel electrophoresis. Following the standard protocol of the ALFA-SEQ DNA Library Prep Kit (Ark Biosafety Technology (Guangzhou) Co., Ltd., Guangzhou, China), the library construction is carried out. Subsequently, the size of the library fragments is evaluated on the Qsep400 High-Throughput Nucleic Acid and Protein Analysis System (Hangzhou Houze Biotech Co., Ltd., Hangzhou, China), and the concentration of the library is measured using the Qubit 4.0 Fluorometer (Thermo Fisher Scientific, Waltham, MA, USA). The library was sequenced on the Illumina NovaSeq 6000 platform, and 250 bp paired-end (PE) reads were generated (Guangdong Magigene Biotechnology Co. Ltd., Guangdong, China).

### 2.6. LC-MS Conditions for Metabolomics Analysis

The metabolomics analysis of the liver sample was conducted using an ultra-performance liquid chromatography (UPLC) system (Vanquish, Thermo Fisher Scientific, Waltham, MA, USA). An ACQUITY UPLC BEH Amide column (50 mm × 2.1 mm, 1.7 µm, Waters, UK) was used for the separation. The mobile phase A of liquid chromatography is an aqueous phase containing 25 mmol/L ammonium acetate and 25 mmol/L ammonia water, while mobile phase B is acetonitrile. Sample tray temperature is 4 °C, and injection volume is 2 μL. The Orbitrap Exploris 120 mass spectrometer (Thermo Fisher Scientific, Waltham, MA, USA) was used to detect the eluted metabolites. The Orbitrap Exploris 120 mass spectrometer was operated in both positive and negative ion modes. Sheath gas flow rate was 50 Arb, Aux gas flow rate was 15 Arb, capillary temperature was 320 °C, full MS resolution was 60,000, MS/MS resolution was 15,000, collision energy was SNCE 20/30/40, spray voltage was 3.8 kV (positive) or −3.4 kV (negative), respectively. During the entire acquisition period, the mass accuracy was calibrated after every 20 samples. Furthermore, a quality control (QC) sample was analyzed after every 10 samples to evaluate the stability of LC-MS during the whole acquisition (for relevant details, refer to Supplementary Materials).

### 2.7. 16S rRNA Data Analysis

Paired-end (PE) reads were assigned to individual samples based on their unique barcode sequences, which were subsequently truncated to remove the barcode and primer sequences. The resulting reads were merged using the FLASH v1.2.11 software. Quality filtering was performed under stringent conditions using the fastp (v0.14.1) to obtain high-quality clean tags (For QC, refer to Supplementary Materials Table S2). Chimeric sequences were identified and removed using the Usearch software (v10.0.240). DADA2 was employed for dereplication, resulting in a feature table and sequence data. Feature abundance was normalized relative to each sample’s total abundance based on the SILVA (release 132) classifier. Operational taxonomic units (OTUs) were analyzed at a 97% sequence similarity threshold using the Usearch (v10.0.240). Alpha-diversity indices, including ACE, coverage, Simpson, Chao1, and Shannon_e, were calculated using the QIIME2 2023.5 to evaluate species complexity. Beta diversity was assessed through principal coordinate analysis (PCoA) and non-metric multidimensional scaling (NMDS) based on bray_curtis, also using QIIME2. Differential intestinal microbiota between groups were detected, and their species composition was analyzed at the phylum and genus levels. Linear discriminant analysis effect size (LEfSe) analysis was performed to identify differential microbiota taxa. Linear discriminant analysis effect size (LEfSe) was performed based on the OTU annotation results, and multiple testing correction was conducted using Benjamini–Hochberg FDR (*p* < 0.05) to identify species with significant differences in abundance between groups. Differential species analysis was conducted using the non-parametric Kruskal–Wallis rank-sum test and Wilcoxon rank-sum test. Bacterial genera with linear discriminant analysis (LDA) score greater than 3 and *p*-values less than 0.05 were selected for further analysis, aiming to discover the differential biomarker species among the intestinal microbiota of tilapia under different treatments. All statistical analyses were conducted using R version 4.3.3 and different R packages (phyloseq v1.42.0, DESeq2 v1.38.3, vegan v2.6.2 and ggplot2 v3.3.6).

### 2.8. Metabolomics Data Processing

The raw data were converted to the mzXML format using ProteoWizard 3.0.7414 and processed with an in-house program, which was developed using R and based on XCMS, for peak detection, extraction, alignment, and integration. Then, the MS2 database was applied to metabolite annotation. The cutoff for annotation was set at 0.3. Using the R package stats (v4.0.3), principal component analysis (PCA) is conducted. For Orthogonal Projections to Latent Structures–Discriminant Analysis (OPLS-DA), the R language package ropls (v1.22.0) is employed. Variable Importance in the Projection (VIP) scores > 1, along with absolute Fold Change (FC) values > 2 or <0.5, and *p* < 0.05, are applied as criteria for screening differential metabolites. The selected differential metabolites are then annotated, classified, clustered, and analyzed using the proprietary database established by Guangdong Magigene Biotechnology Co., Ltd. Venn diagrams are generated using Metware Cloud 2024.1.26 (https://cloud.metware.cn; accessed on 24 March 2024) for visualizing and comparing sets of differential metabolites or pathways across different experimental conditions or analyses. Metabolic pathway analysis is performed via MetaboAnalyst 6.0 (https://genap.metaboanalyst.ca/; accessed on 26 March 2024), with statistical significance set at *p* < 0.05. Metabolic pathways with an impact value > 0.1 are considered significantly relevant to the study conditions, indicating that these pathways are the most pertinent ones associated with the experimental conditions. The correlation heatmap was drawn using the OmicStudio tools 3.6 at https://www.omicstudio.cn/tool (accessed on 8 October 2024).

### 2.9. Statistical Analysis

All data are presented as means ± SD. The statistical analysis was carried out with SPSS 18.0 statistical software (SPSS Inc., Chicago, IL, USA). *p* ≤ 0.05 was considered significant. Spearman’s correlation analysis was conducted to analyze the correlation relationship between the microbiota and metabolite using the data of significantly differential genus-level microbiota and differential secondary metabolites. A scaled heatmap was constructed for the correlation matrix using the default clustering method.

## 3. Results

### 3.1. Xanthohumol Inhibits SA Replication in Tilapia

**Figure 1:**
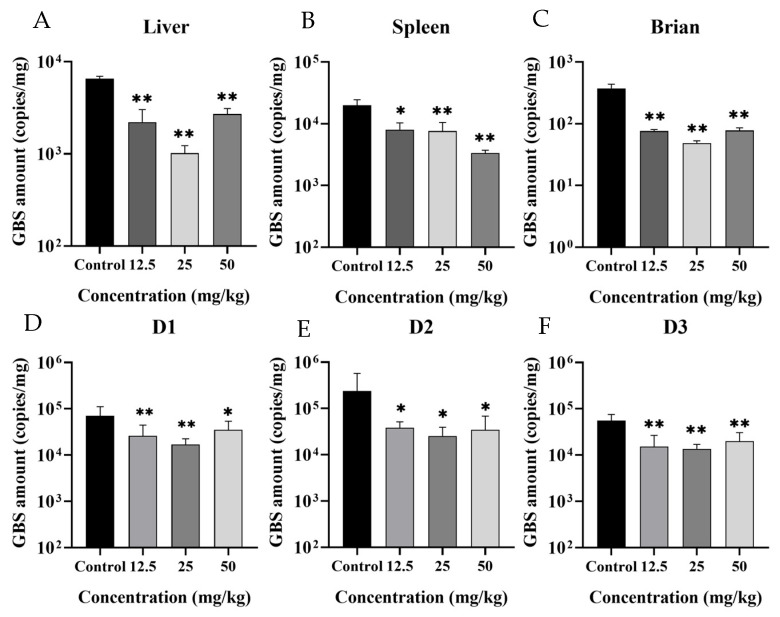
Xanthohumol (XN) inhibited the proliferation of Streptococcus agalactiae (SA) in tilapia. Bacterial loads in the (**A**) liver, (**B**) spleen and (**C**) brain of SA-infected tilapia treated with different concentrations of XN for 24 h. Bacterial loads in the liver of SA-infected tilapia treated with different concentrations of XN for (**D**) 24 h, (**E**) 48 h and (**F**) 72 h. Asterisks mark the significant difference between experimental data and control data (* *p* < 0.05, ** *p* < 0.01).

As shown in Figure 1A–C, XN could significantly inhibit SA infection in different tissues of tilapia after treatment with different concentrations of XN for 24 h. Compared with the control groups, the copy numbers of SA in the liver, spleen, and brain tissues of tilapia in the XN groups reduced by 6.45-fold, 5.96-fold and 7.73-fold, respectively. Furthermore, XN could also significantly inhibit SA infection in the liver tissues of tilapia after treatment with different concentrations of XN for 24, 48 and 72 h (Figure 1D–F).

### 3.2. Xanthohumol Improves the Survival Rate of SA-Infected Tilapia

**Figure 2:**
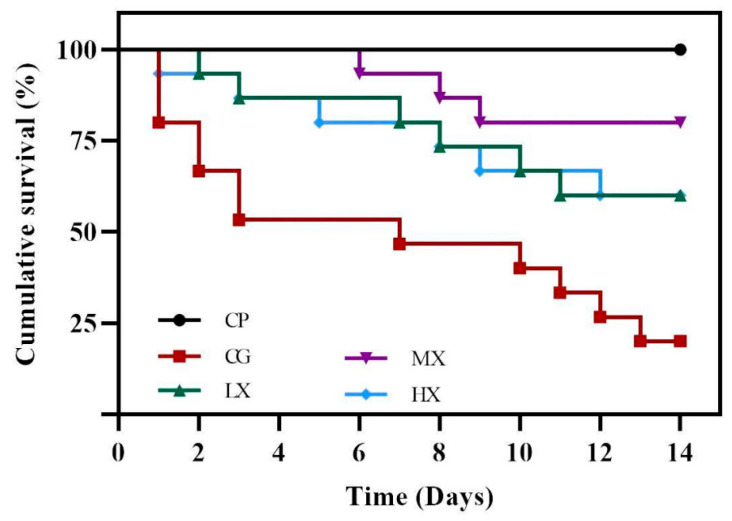
XN improved the survival rate of tilapia infected with SA. The CP groups were injected with PBS solution only; the CG groups were injected with SA bacterial solution only; the LX (12.5 mg/kg XN), MX (25 mg/kg), and HX (50 mg/kg XN) groups were injected with a mixture of XN and SA bacterial solution. The mortality of each group (*n* = 45) was continuously detected for 14 days. Survival Rate = (Number of Survivors/Initial Number) × 100%.

To explore the therapeutic effect of XN, SA-infected tilapia was treated with XN (12.5, 25, and 50 mg/kg) and observed for 14 days. As shown in Figure 2, the survival rate of tilapia was significantly different between the treatment and control groups. The cumulative survival rate of tilapia in the CP groups (treated with PBS only) was 100%, while it was only 20% in the CG groups (treated with SA only). Interestingly, the cumulative survival rates of SA-infected tilapia after treatment with 12.5 mg/kg (LX groups), 25 mg/kg (MX groups) and 50 mg/kg (HX groups) XN for 14 d were increased to 40%, 80% and 40%, respectively.

### 3.3. Effects of Xanthohumol on the Intestinal Microbial Diversity

**Figure 3:**
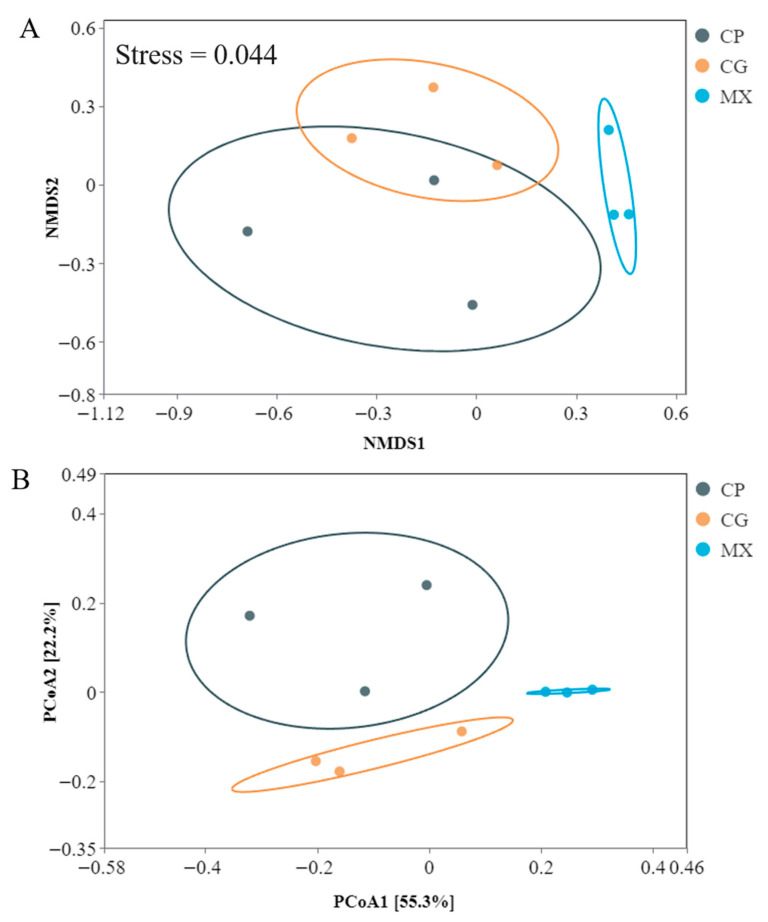
The β-diversity of intestinal microbiota of tilapia in different groups. (**A**) NMDS analysis. Stress = 0.044 indicates that NMDS can accurately reflect changes in the community between samples; (**B**) PCoA analysis. PCoA scatter plot of OTUs. The gray, yellow and blue circles represent the CP group, CG group and MX group, respectively.

As shown in Table 1, there were no significant differences in ACE and coverage indices among the MX groups, CP groups and CG groups (*p* > 0.05), but the ACE index in the MX groups was higher than that in the CG groups, and the MX groups were lower than that in the CP groups. There were significant differences in Simpson, Chao1, and Shannon_e indices between both the MX group and CG groups, as well as between the CP group and CG groups (*p* < 0.05). The results of the α-diversity indices indicated that XN could regulate the α-diversity of intestinal microbial communities in tilapia. In addition, the Bray–Curtis similarity analysis of NMDS and PCoA among different groups showed that there were significant differences in microbial composition among groups (Figure 3).

### 3.4. Effects of Xanthohumol on Intestinal Microbial Composition

**Figure 4:**
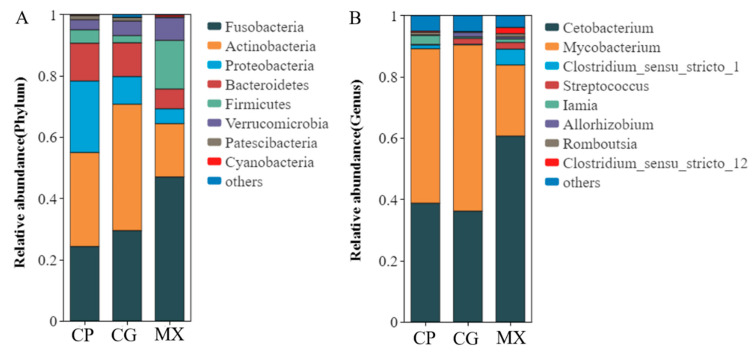
The relative abundance of intestinal microbiota in different groups at the phylum (**A**) and genus (**B**) taxonomic level.

As shown in Figure 4, the intestinal microbial composition of the dominant microbiota was essentially the same but with changes in abundance. At the phylum level, the relative abundance of the phyla Actinobacteria, Proteobacteria and Bacteroidetes decreased in the MX compared to the CG, while the relative abundance of the phyla Fusobacteria, Firmicutes and Verrucomicrobia increased (Figure 4A). Compared with the CP group, the relative abundance of Proteobacteria, Bacteroidetes, and Firmicutes in the CG group was decreased, while the relative abundance of Fusobacteria, Actinobacteria, and Verrucomicrobia was increased (Figure 4A). Differences were also observed at the genus level; the relative abundance of *Mycobacterium* decreased in the MX, but the abundance of *Cetobacterium* and *Clostridium_sensu_stricto_1* increased (Figure 4B). Compared with the CP group, the relative abundance of Cetobacterium and Clostridium_sensu_stricto_1 in the CG group was decreased, while the abundance of Mycobacterium was increased (Figure 4B).

### 3.5. Identification of Potential Intestinal Microbial Biomarkers

To evaluate the potential biomarker taxa in tilapia, we compared the intestinal microbial communities between the CG and CP groups and the CG and MX groups using the linear discriminant analysis effect size (LEfSe, Table 2). LEfSe analysis was performed based on the LDA effect size and could be used to compare between groups for the identification of biomarker taxa with the most significant differences in abundance. Between the CG and CP groups, the CP groups had four taxa with significantly enriched abundance, while three taxa were upregulated in the CG groups. In addition, the MX groups had 17 taxa with significantly enriched abundance compared to the CG groups, and 5 taxa were upregulated in the CG groups. In CG vs. MX, the biomarkers recognized for the MX group were *g_Cetobacterium* and *g_Clostridium_sensu_stricto_1*, while *g_Mycobacterium* was identified as biomarkers in the CG group.

### 3.6. Effect of Xanthohumol on Metabolomic Alterations

**Figure 5:**
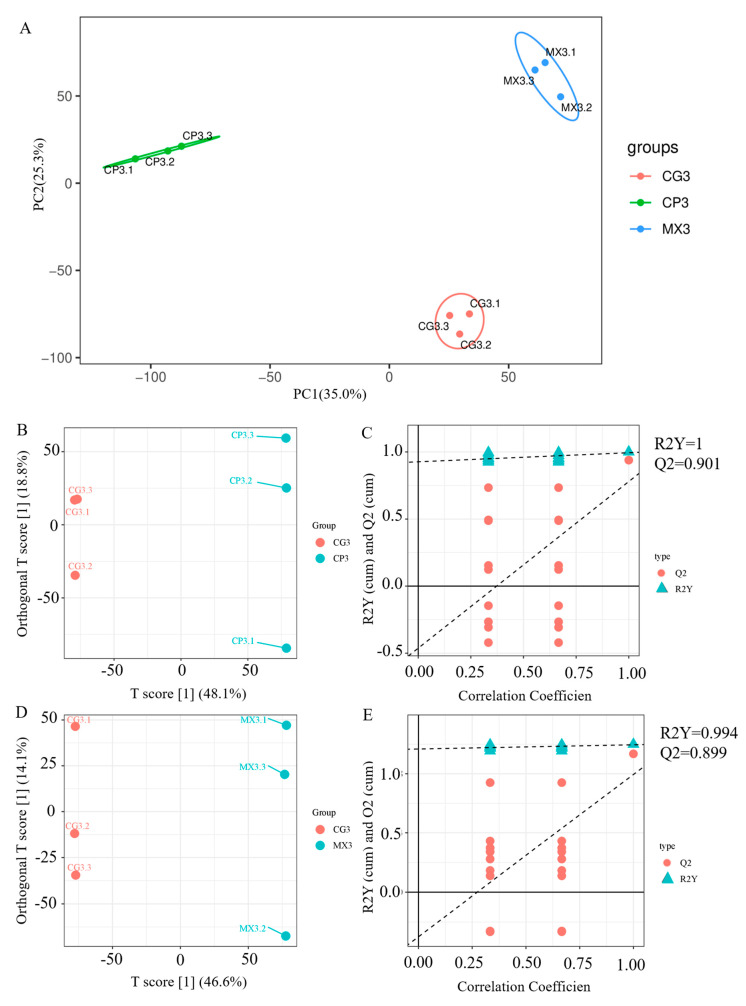
The PCA and OPLS-DA analysis of liver metabolites of tilapia in different groups. (**A**) PCA analysis. The yellow, green and blue circles represent the CG3 group, CP3 group and MX3 group, respectively; (**B**) score scatter plot and (**C**) permutation test result plot of OPLS-DA model in CG3 vs. CP3; (**D**) score scatter plot and (**E**) permutation test result plot of OPLS-DA model in MX3 vs. CG3.

The results of PCA analysis showed that there was no overlap between CP3 and CG3 and CG3 and MX3 (Figure 5A). Figure 5B–E show scatter plots of the OPLS-DA model between the CG and CP groups and CG and MX groups. The farther the distance between the points, the greater the difference between their physiological and pathological states. It can be seen from Figure 5B,D that the main components of the CG3 group and CP3 group and the CG3 group and MX3 group are separated and do not overlap; thus, there is a significant difference between the CG3 group and CP3 group and the CG3 group and MX3 group. Meanwhile, we use the OPLS-DA model to calculate the VIP through the control group to assess the contribution of metabolites to the differences between groups. To assess the model, an alignment test is performed. An alignment verification chart is shown in Figure 5. As shown in Figure 5C,E, the original model has a good fit and there is no overfitting.

### 3.7. Differential Metabolic Pathways and Differential Metabolites

**Figure 6:**
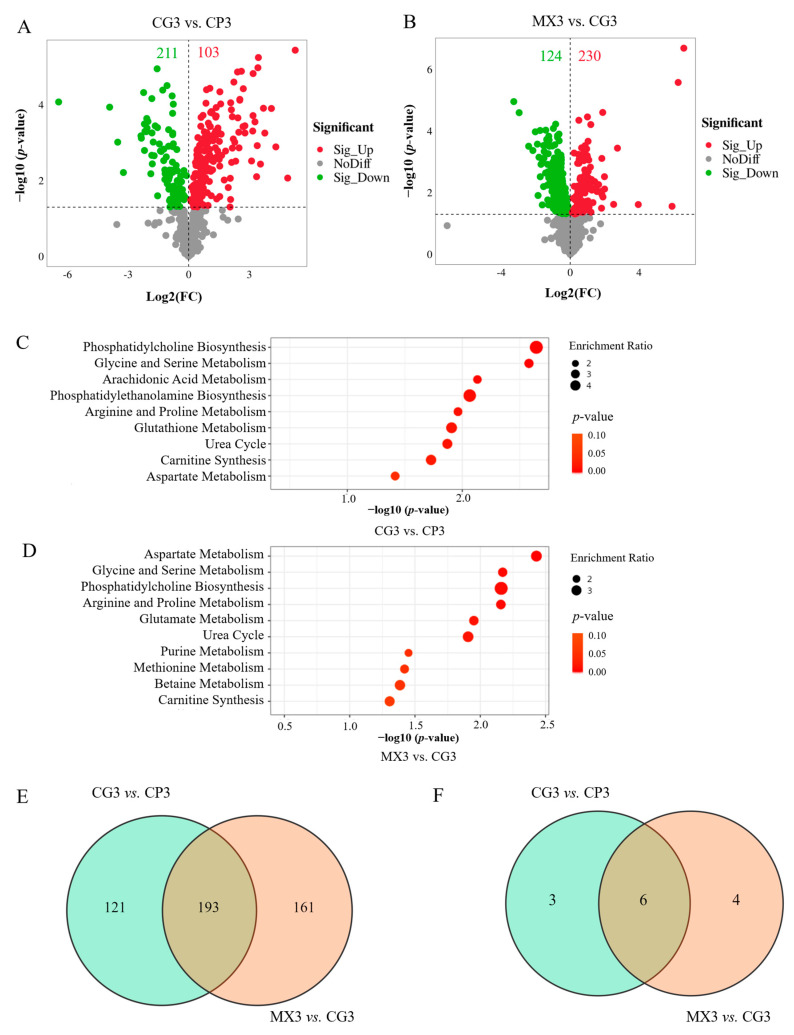
The analysis of DEMs and differential metabolic pathways in different groups. The volcano plot of DEMs in CG3 vs. CP3 (**A**) and in MX3 vs. CG3 (**B**); the pairwise comparison of differential metabolic pathways in CG3 vs. CP3 (**C**) and MX3 vs. CG3 (**D**); the Venn diagram of DEMs (**E**) and differential metabolic pathways (**F**) in different groups.

Compared with the CP3 group, there were 211 downregulated and 103 upregulated metabolites in the CG3 group (Figure 6A). Compared with the CG3 group, the number of downregulated and upregulated metabolites in the MX3 group was 124 and 230 (Figure 6B), respectively.

Differential metabolites were imported into the Metaboanalyst 6.0 database for metabolic pathway analysis. The results of the differential metabolic pathways indicated that, in CG3 vs. CP3, the metabolic pathways of phosphatidylcholine biosynthesis, glycine and serine metabolism, arachidonic acid metabolism, phosphatidylethanolamine biosynthesis, arginine and proline metabolism, glutathione metabolism, urea cycle, carnitine synthesis, aspartate metabolism in the CG3 group were mainly enriched (Figure 6C). In MX3 vs. CG3, the metabolic pathways of aspartate metabolism, glycine and serine metabolism, phosphatidylcholine biosynthesis, arginine and proline metabolism, glutamate metabolism, urea cycle, purine metabolism, methionine metabolism, betaine metabolism, carnitine synthesis in the MX3 group were mainly enriched (Figure 6D).

The Venn diagram analysis revealed an overlap of 193 differentially expressed metabolites (DEMs) and 6 differential metabolic pathways shared between the two comparison groups (Figure 6E,F). Additionally, exclusive to the CG3 versus CP3 comparison, there were 121 unique DEMs and 3 distinct metabolic pathways, whereas the MX3 versus CG3 comparison exhibited 161 exclusive DEMs and 4 unique metabolic pathways.

### 3.8. Association Between the Intestinal Microbiota and Metabolites

**Figure 7:**
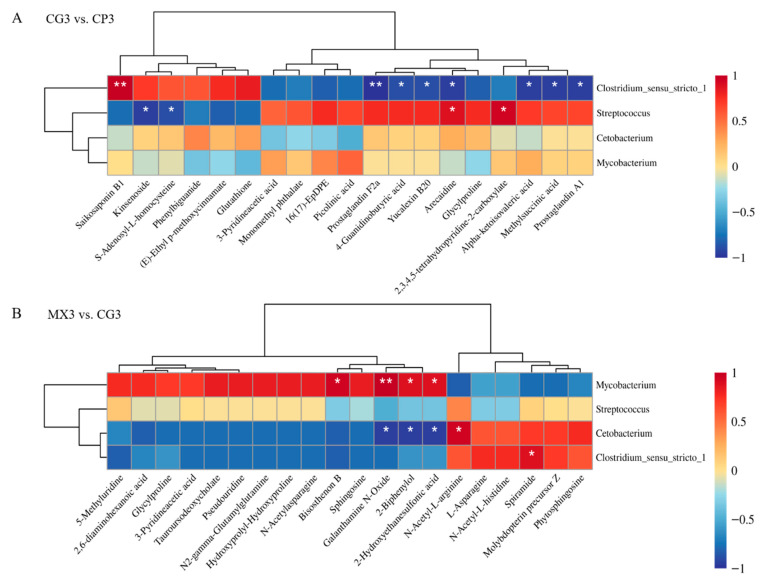
The combination analysis of differential microbiota and differential metabolites in pairwise comparison of the three groups. (**A**) CG3 vs. CP3, (**B**) MX3 vs. CG3 (* *p* < 0.05, ** *p* < 0.01).

The correlation analysis of 16S sequencing and metabolomics was performed to explore the relationship between the intestinal microbiota and host metabolism using the data of differential genus-level microbiota and differential secondary metabolites using Pearson’s correlation analysis (Figure 7). According to the genus-level microbial species analysis, compared with the other two groups, the relative abundance of *cetobacterium* and *Clostridium_sensu_stricto_1* in the MX group was higher, while the relative abundance of *Mycobacterium* was lower. As shown in Figure 7A, Spearman’s correlation analysis revealed that in the comparison between CG3 and CP3, *Clostridium_sensu_stricto_1* exhibited a significant positive correlation with saikosaponin B1. In contrast, prostaglandin F2a, 4-guanidinobutyric acid, Yucalexin B20, arecaidine, alpha-ketoisovaleric acid, methylsuccinic acid, and prostaglandin A1 showed significant negative correlations with *Clostridium_sensu_stricto_1*. Moreover, *Streptococcus* had a significant positive correlation with arecaidine and 2,3,4,5-tetrahydropyridine-2-carboxylate, while kinsenoside and S-Adenosyl-L-homocysteine showed significant negative correlations with *Streptococcus*. As shown in Figure 7B, in the comparison between MX3 and CG3, *Clostridium_sensu_stricto_1* demonstrated a significant positive correlation with sphingosine. *Mycobacterium* showed a significant positive correlation with bisosthenon B, galanthamine N-Oxide, 2-Biphenylol, and 2-Hydroxyethanesulfonic acid. *Cetobacterium* also had a significant positive correlation with galanthamine N-Oxide, 2-Biphenylol, and 2-hydroxyethanesulfonic acid, whereas N-Acetyl-L-arginine showed a significant negative correlation with *Cetobacterium*. However, the correlation analysis in this study only reveals statistical associations between variables, and causal relationships cannot be inferred at this stage.

## 4. Discussion

In the present study, we utilized qPCR to assess the variation in SA loads in the tissues of tilapia following SA infection and subsequent treatment with XN. This approach was employed to quantify the drug’s anti-SA efficacy and to evaluate its potential as an anti-SA agent. In disease treatment, the effectiveness of a drug depends on the dosage, and many phytochemicals exhibit hormetic effects. This is a biphasic dose–response model, usually showing a “J”-shaped or inverted “U”-shaped curve, which is characterized by “stimulation at low doses and inhibition at high doses” [13]. In our study, different concentrations of XN all showed significant protective effects on tilapia, among which the 25 mg/kg concentration exhibited the best efficacy. This is highly consistent with the hormetic effect model. Furthermore, our survival rate results were similar to the anti-SA effects, where the 25 mg/kg XN exhibited the optimal protective effect, further supporting that xanthohumol may possess a hormetic effect. In other studies, 5 mg/L XN can alleviate DNA damage caused by oxidative stress in *Saccharomyces cerevisiae*, while concentrations higher than 5 mg/L lead to a reduced growth rate and aggravated DNA damage [14]. In summary, XN may exhibit a hormetic effect and has a good anti-SA effect in tilapia, indicating that XN has great therapeutic potential for SA infection.

The integrity of gut microbiota structure and function is intricately linked to microbial diversity [15], which, in turn, is correlated with the health status of fish [16]. In alignment with previous findings, SA infection significantly diminishes the richness and diversity of the zebrafish gut microbiota while concurrently increasing the prevalence of pathogenic bacteria, thus aggravating the zebrafish condition [17]. The diversity resistance hypothesis posits that microbial communities with greater diversity are more likely to contain species capable of resisting invaders or pathogens [18]. Treatment with XN has been shown to elevate the ACE, Chao, and Shannon indices of the tilapia gut microbiota and to decrease the Simpson index, suggesting an enhancement in α-diversity. The reason for this phenomenon may be that, although XN has antibacterial activity, it mainly targets and inhibits pathogenic bacteria while having little impact on beneficial bacteria in the intestine. In addition, XN may promote the proliferation of beneficial bacteria by regulating intestinal composition and improving the intestinal environment, thereby increasing the overall diversity of microbiota. This mechanism is different from that of traditional broad-spectrum antibiotics, reflecting the specific advantages of XN. This implies that XN may bolster resistance to SA invasion through the promotion of gut microbial α-diversity.

The bacterial community composition in the XN-treated group diverged from that of the infected group, suggesting that both XN and SA are capable of modulating the tilapia gut microbiota structure. Donoso et al. [19] observed that XN can alter the abundance of specific gut microbial genera and significantly affect the β-diversity in a rat maternal separation model. Logan et al. [20] discovered through 16S rRNA gene sequencing of fecal samples from obese mice that XN supplementation can modify the microbial composition and substantially alter the predicted functional capabilities of the gut microbiota. Additionally, dietary garlic supplementation has been shown to regulate the gut microbial community in tilapia, influencing bacterial counts, diversity indices, and community structure, thereby countering *Streptococcus iniae* infection [21]. These findings collectively suggest that XN may alter the tilapia gut microbiota structure, potentially conferring protection against SA infection.

In the XN-treated group, the relative abundance of *Cetobacterium* and *Clostridium_sensu_stricto_1* was significantly higher compared to the infected group, whereas *Mycobacterium* was significantly less abundant. Li Ming et al. [22] noted significant shifts in the gut microbiota composition and diversity of tilapia following oral SA infection, with an increase in pathogenic genera such as *Edwardsiella* and *Pseudomonas* and a decrease in beneficial genera like *Cetobacterium*, *Lactococcus*, and *Propionibacterium*. Zhao et al. [23] reported a marked increase in the relative abundance of the pathogen *Campylobacter* in the gut of SA-infected tilapia under high-temperature conditions, potentially compromising the fish’s immune defenses. *Mycobacterium* is a genus known to pose significant threats to fish, with several species inducing severe and often fatal diseases across a broad spectrum of fish species globally [24,25]. *Cetobacterium*, an anaerobic bacterium native to the guts of many freshwater fish, has been shown to produce butyrate through the fermentation of peptides and carbohydrates, thereby inhibiting the growth of potential pathogens [26]. Recent studies have highlighted *Cetobacterium*’s ability to produce vitamin B12, which fortifies intra-microbiota interactions and enhances the host’s resistance to pathogenic infections [27]. Consistent with our observations, Radix Rehmanniae Preparata polysaccharides have also been reported to increase the abundance of *Cetobacterium* in the perch gut [28]. *Clostridium_sensu_stricto_1*, capable of metabolizing proteins and carbohydrates to produce small organic acids and hydrogen, is regarded as a beneficial bacterial genus in the context of bacterial infections due to its butyrate production [29]. XN can specifically enrich *Clostridium_sensu_stricto_1* and *Cetobacterium* (which are capable of producing short-chain fatty acids) in the intestines of tilapia, whereas other plant extracts (such as allicin) mainly exert their effects directly by inhibiting pathogenic bacteria [30,31]. This unique mode of action may be an important reason for the in vivo antibacterial activity of XN. This indicates that XN may counteract SA infection by increasing the proportion of beneficial bacteria, such as *Cetobacterium* and *Clostridium_sensu_stricto_1*. However, regarding the impact of XN on the intestinal microbiota of tilapia, due to taxonomic limitations, further verification of its role in the tilapia intestine through metagenomics or single-bacterium isolation and culture experiments will be required in the future.

Bacterial infections are a significant cause of metabolic disorders in fish. Current research indicates that a variety of bacteria, including *Vibrio vulnificus*, *Vibrio alginolyticus*, *Aeromonas hydrophila*, *Edwardsiella tarda*, and SA, can alter the host’s metabolic pathways [32,33,34,35,36]. The findings suggest that the metabolic state of aquatic animals is closely related to their infection resistance. For instance, studies have shown that *crucian carp* can regulate their metabolism to resist *Edwardsiella tarda* infection at 30 °C [37]. Existing research indicates that XN can ameliorate the dysfunctional glucose and lipid metabolism in diet-induced obese mouse models [20]. Our results indicated that, compared with the CG3 group, the number of downregulated and upregulated metabolites in the MX3 group was 124 and 230. Moreover, many metabolic pathways, including the aspartate metabolism, glycine and serine metabolism and phosphatidylcholine biosynthesis, were enriched in the MX3 group, indicating that XN changed the tilapia metabolic profiles. Aspartate was found to enhance the defense capability of zebrafish against antibiotic-sensitive and -resistant *Edwardsiella tarda* by promoting the biosynthesis of nitric oxide (NO) [38]. Exogenous aspartate could protect zebrafish from infection, and similarly, exogenous aspartate could enable *Cyprinus carpio* to combat *Aeromonas hydrophila* infection by promoting the release of NO [35]. Furthermore, aspartate metabolism also promotes the secretion of interleukin-1β (IL-1β) in inflammatory macrophages [39], indicating that aspartate metabolism also plays a key role in regulating macrophage function and inflammatory responses. Glycine and serine are two non-essential amino acids that play significant roles in the growth, health, and immune systems of aquatic animals. Glycine is involved in the construction of the antioxidant system and can stimulate the immune system [40]. In *Cyprinus carpio*, dietary supplementation with glycine can significantly improve growth performance, red blood cell stability, and humoral and mucosal immunity [40]. The regulation of serine metabolism can promote the survival of Nile tilapia after infection with *Edwardsiella tarda* [36].

The gut microbiota plays a key role both in the digestion and absorption of amino acids and in the breakdown and fermentation of amino acids in the intestine [41]. The intestinal microbiota of tilapia is rich in species participating in amino acid metabolism [42], especially, *Cetobacterium* and *Clostridium_sensu_stricto_1* [30]. The Spearman correlation analysis indicates a positive correlation between *Cetobacterium* and *Clostridium_sensu_stricto_1* with N-Acetyl-L-arginine, L-Asparagine, and N-Acetyl-L-histidine. Thus, XN may have enhanced the number of microorganisms capable of producing metabolically produced amino acids to facilitate immunopotentiation in the host.

**Figure 8:**
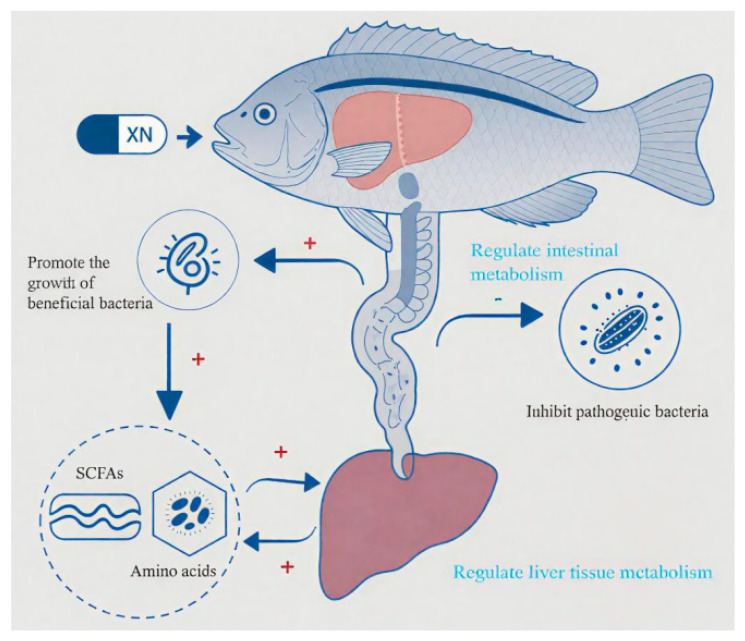
XN-mediated modulation of microbiota and liver metabolism in preventing SA infection. “+” represents promotion, and “−“ represents inhibition.

XN shows promise as a preventive and therapeutic agent against SA infection in tilapia, with its mechanism of action likely involving the regulation of intestinal microbiota and liver metabolism (Figure 8). This study provides a foundation for the application of natural products in aquaculture to combat bacterial infections.

## Figures and Tables

**Table 1:** The α-diversity analyses of intestinal microbiota in the three groups.

Index	CG	CP	MX
ACE	430.88 ± 17.10	473.79 ± 25.22	464.80 ± 15.92
Coverage	1.00 ± 0.00	1.00 ± 0.00	1.00 ± 0.00
Simpson	0.26 ± 0.02 ^a^	0.18 ± 0.01 ^b^	0.15 ± 0.01 ^c^
Chao1	265.63 ± 14.25 ^b^	321.63 ± 21.47 ^a^	346.20 ± 10.85 ^a^
Shannon_e	2.08 ± 0.08 ^b^	2.40 ± 0.07 ^ab^	2.55 ± 0.06 ^a^

Different letters in the same row indicate the significant differences between various groups (*p* < 0.05).

**Table 2:** LEfSe analysis of group CP, group CG and group MX at the genus level.

	Group	Biomarker ID	LDA	*p*-Value
CG vs. CP	CG	*g. Allorhizobium*	3.654	0.0495
		*g. Streptococcus*	3.552	0.0495
		*g. Brevinema*	3.219	0.0495
	CP	*g. Iamia*	3.789	0.0495
		*g. Clostridium_sensu_stricto_1*	3.424	0.0495
		*g. Romboutsia*	3.207	0.0495
		*g. Anaerobacterium*	3.092	0.0463
CG vs. MX	CG	*g. Deinococcus*	3.990	0.0369
		*g. Anoxybacillus*	3.879	0.0463
		*g. Methylobacterium*	3.388	0.0495
		*g. Mycobacterium*	3.042	0.0369
		*g. Xanthobacter*	3.004	0.0463
	MX	*g. Cetobacterium*	4.806	0.0495
		*g. Clostridium_sensu_stricto_1*	4.129	0.0495
		*g. Lachnoclostridium*	4.090	0.0369
		*g. Massilia*	3.961	0.0369
		*g. Clostridium_sensu_stricto_2*	3.895	0.0463
		*g. Ruminococcaceae_UCG*	3.762	0.0495
		*g. Caproiciproducens*	3.650	0.0369
		*g. Clostridium_sensu_stricto_12*	3.646	0.0495
		*g. Akkermansia*	3.422	0.0495
		*g. Cellulosilyticum*	3.401	0.0369
		*g. Romboutsia*	3.396	0.0495
		*g. Anaerobacterium*	3.333	0.0463
		*g. Clostridium_sensu_stricto_7*	3.295	0.0463
		*g. Clostridium_sensu_stricto_13*	3.270	0.0495
		*g. Epulopiscium*	3.215	0.0495
		*g. Clostridium_sensu_stricto_18*	3.164	0.0463
		*g. Iamia*	3.144	0.0495

The linear discriminant analysis (LDA) effect size (LEfSe) was used to compare the differences in gut microbial community composition in different groups. LDA score > 3.

## Data Availability

The original contributions presented in this study are included in the article/Supplementary Material. Further inquiries can be directed to the corresponding authors.

## Supplementary Materials

The following supporting information can be downloaded at: https://www.mdpi.com/article/10.3390/microorganisms13071699/s1, Figure S1: Total Ion Chromatogram (TIC) of QC samples detected by UHPLC-OE-MS in positive ion mode; Figure S2: Total Ion Chromatogram (TIC) of QC samples detected by UHPLC-OE-MS in negative ion mode; Figure S3: Extracted Ion Chromatogram (EIC) of internal standards in all QC samples (positive ion mode); Figure S4: Extracted Ion Chromatogram (EIC) of internal standards in all QC samples (negative ion mode); Figure S5: EIC of internal standards in blank and QC samples (positive ion mode); Figure S6: EIC of internal standards in blank and QC samples (negative ion mode); Figure S7: PCA score plot. Green dots represent QC samples, blue dots represent experimental samples; Figure S8: One-dimensional PCA-X distribution of QC samples; Figure S9: Correlation analysis of QC samples; Table S1: Stability of internal standard responses in QC samples; Table S2: QC report on clean tags of 16S rRNA data.
