# Supplementary material for: Effects of Natural Ingredient Xanthohumol on the Intestinal Microbiota, Metabolic Profiles and Disease Resistance to Streptococcus agalactiae in Tilapia Oreochromis niloticus
Source: Microorganisms. 2025 Jul 20;13(7):1699. doi: 10.3390/microorganisms13071699 (PMC12300398; doi:10.3390/microorganisms13071699)
Supplement: Supplementary file 1 [file microorganisms-13-01699-s001.zip › microorganisms-3714328-supplementary.pdf]

Metabolomics quality control

Systematic analysis of TIC chromatograms from QC samples can effectively identify potential issues during experiments, ensuring data reproducibility and comparability. TIC Chromatogram of Experimental QC Samples (Figure S1, Figure S2).

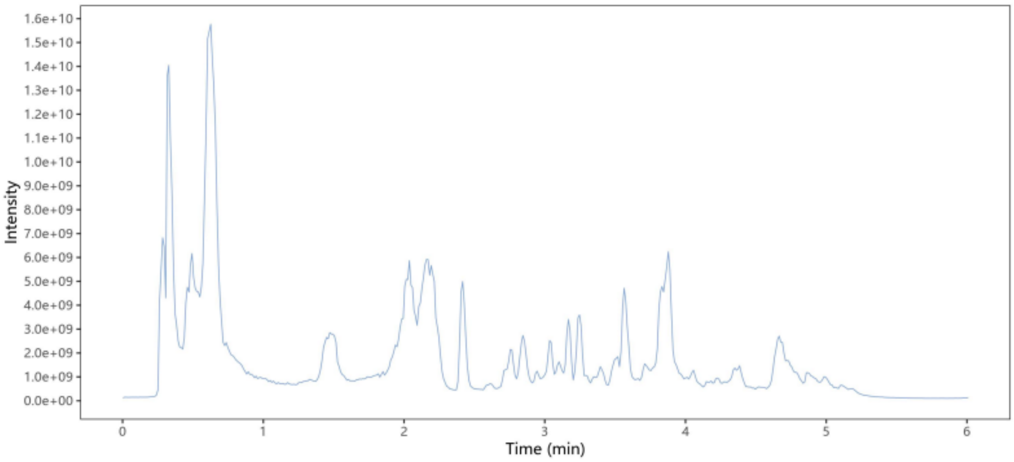

Figure S1. Total Ion Chromatogram (TIC) of QC samples detected by UHPLC-OE-MS in positive ion mode.

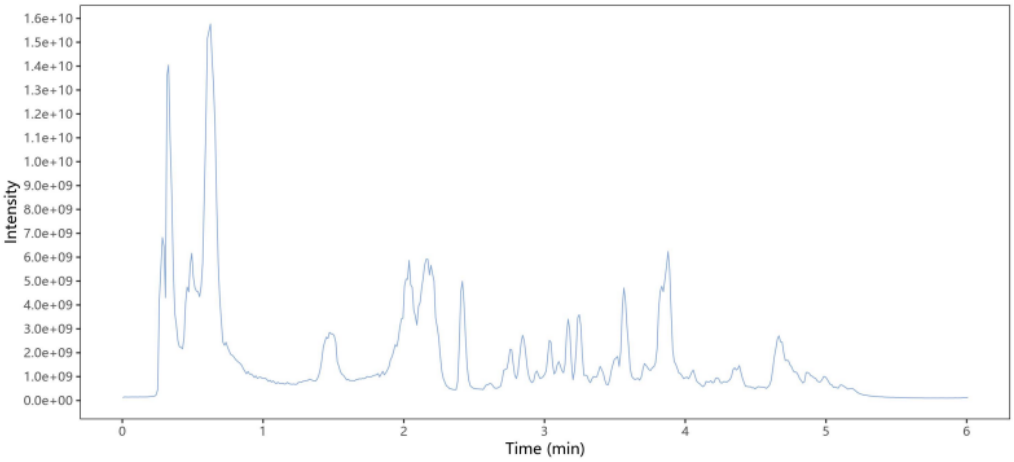

Figure S2. Total Ion Chromatogram (TIC) of QC samples detected by UHPLC-OE-MS in negative ion mode

The stability of the detection system can be evaluated by comparing the peak height differences of internal standards among QC samples. As shown in Figure S3 and Figure S4, the retention times and response intensities of internal standards in QC samples exhibit excellent stability, indicating that the instrument's data acquisition system is highly reliable.

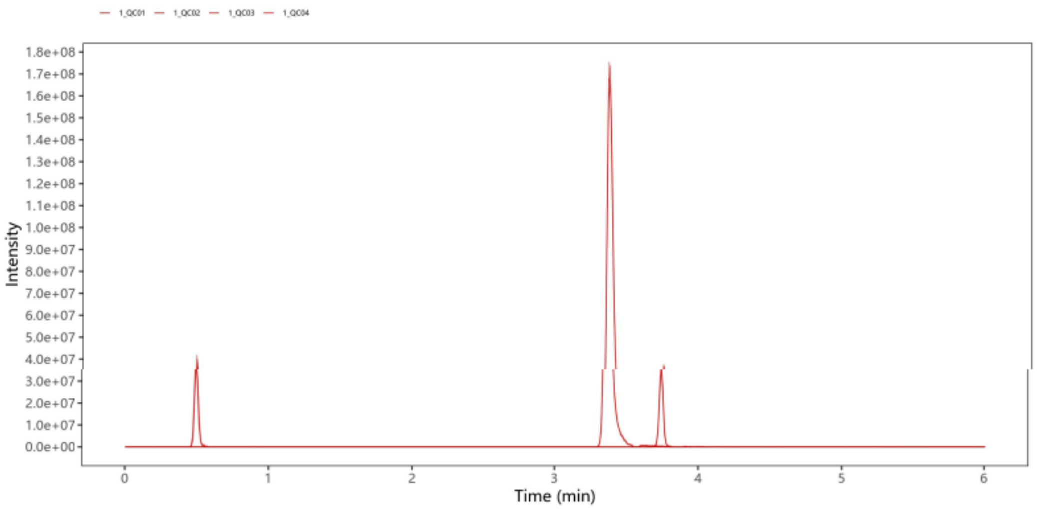

Figure S3. Extracted Ion Chromatogram (EIC) of internal standards in all QC samples (positive ion mode)

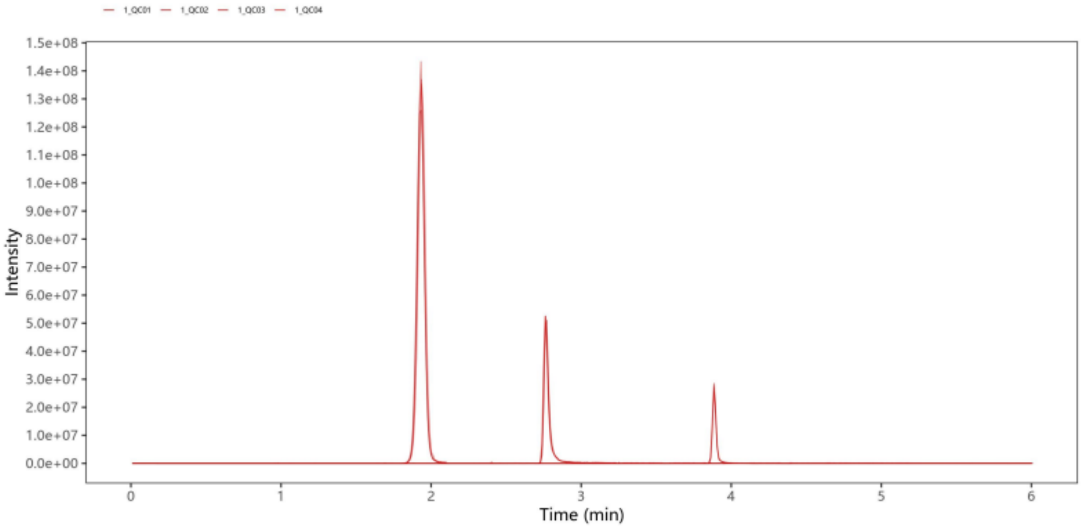

Figure S4. Extracted Ion Chromatogram (EIC) of internal standards in all QC samples (negative ion mode)

The detection of blank samples interspersed throughout the entire experimental process can be used to investigate the residue of substances during the detection process. As can be seen from Fig S5 and S6, no obvious peaks of all internal standards were detected in all blank samples, indicating that the control of substance residues is excellent, and the cross-contamination between samples is within a controllable range.

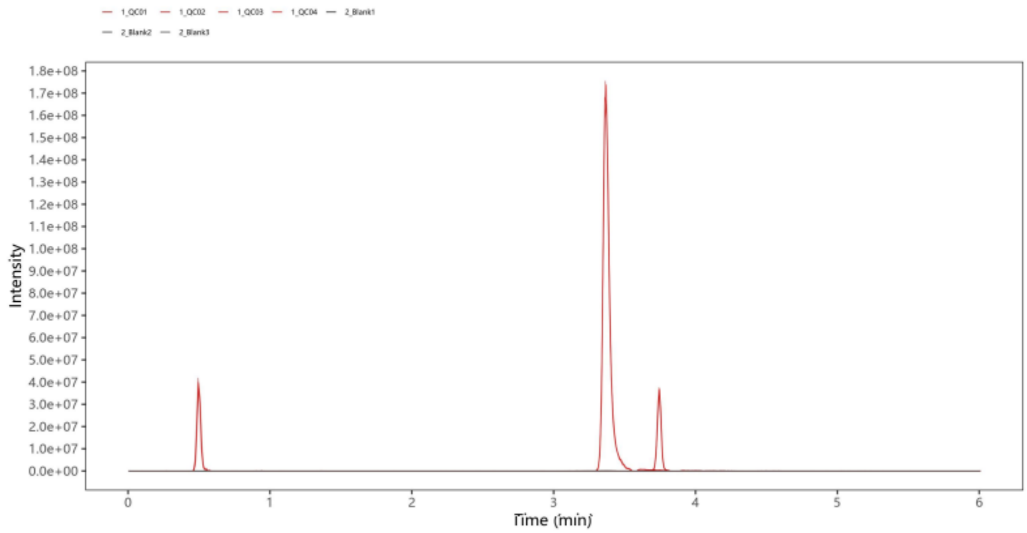

Figure S5. EIC of internal standards in blank and QC samples (positive ion mode)

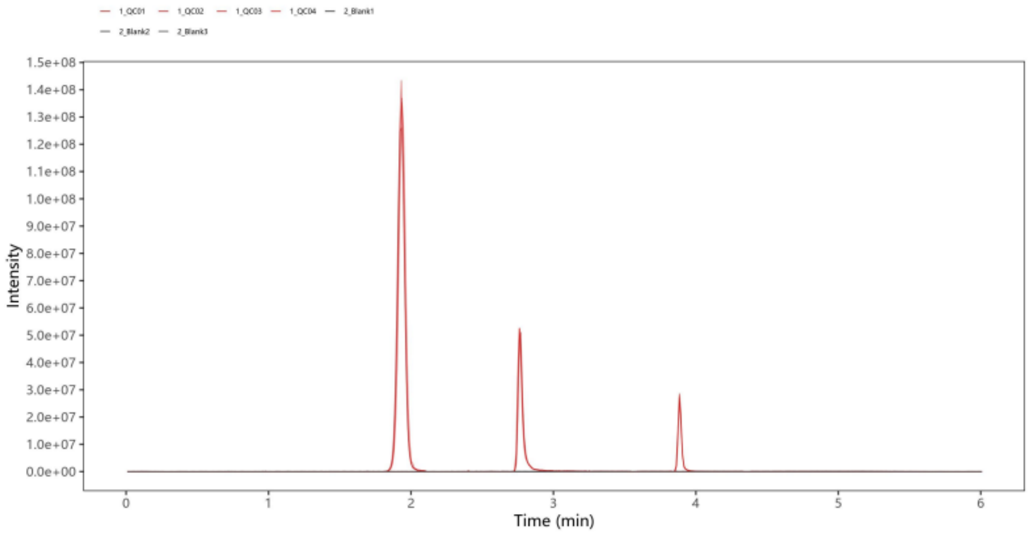

Figure S6. EIC of internal standards in blank and QC samples (negative ion mode)

Theoretically, all QC samples are identical. However, errors may occur during the processes of substance extraction and detection analysis, leading to differences among QC samples. The smaller such differences are, the better the stability of the entire method and the higher the data quality. As can be seen from Fig S7, the QC samples show excellent aggregation, indicating that the method has good stability.

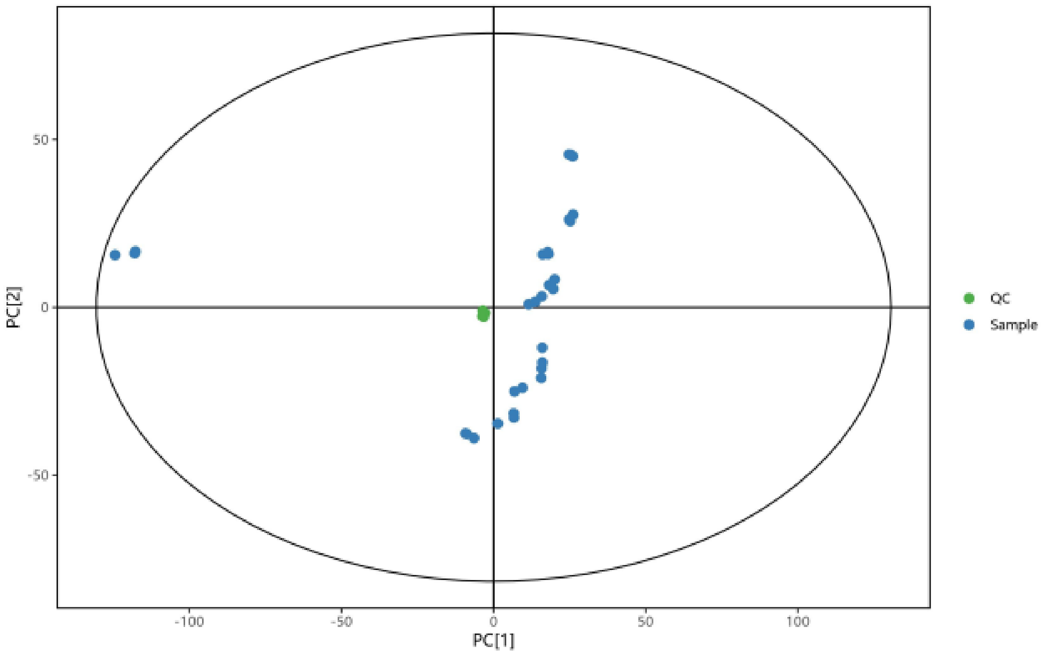

Figure S7. PCA score plot. Green dots represent QC samples, blue dots represent experimental samples.

As can be seen from Figure S8, all QC samples fall within the range of ±2 STD, indicating that the data quality of this experiment is very high.

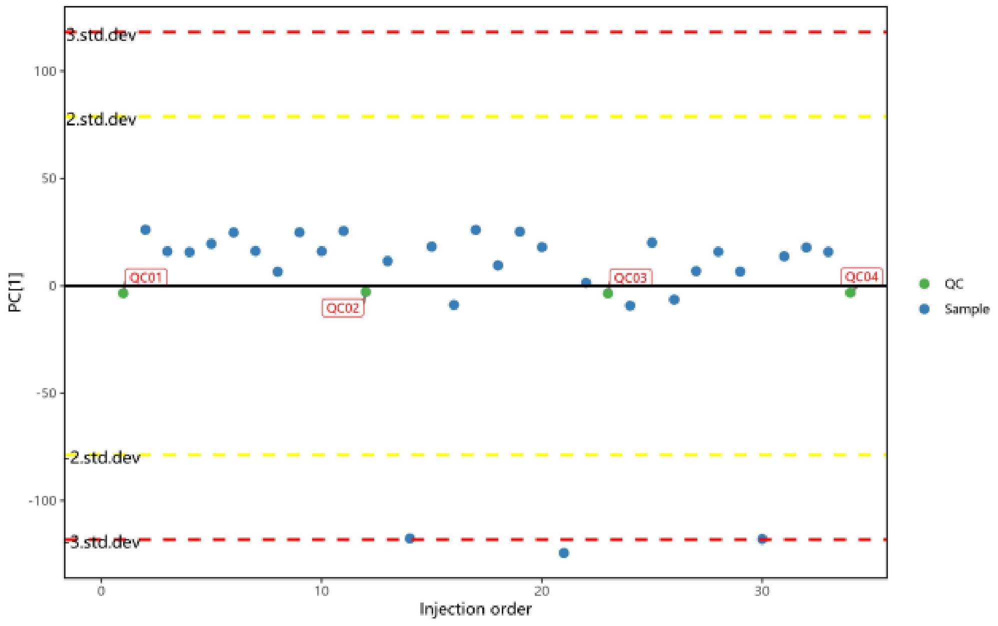

Figure S8. One-dimensional PCA-X distribution of QC samples.

The closer the correlation of QC samples is to 1 (and it should be at least greater than 0.85), the better the stability of the entire method and the higher the data quality. As can be seen from Figure S9, the QC samples have a high correlation, indicating that the data quality of this experiment is very high.

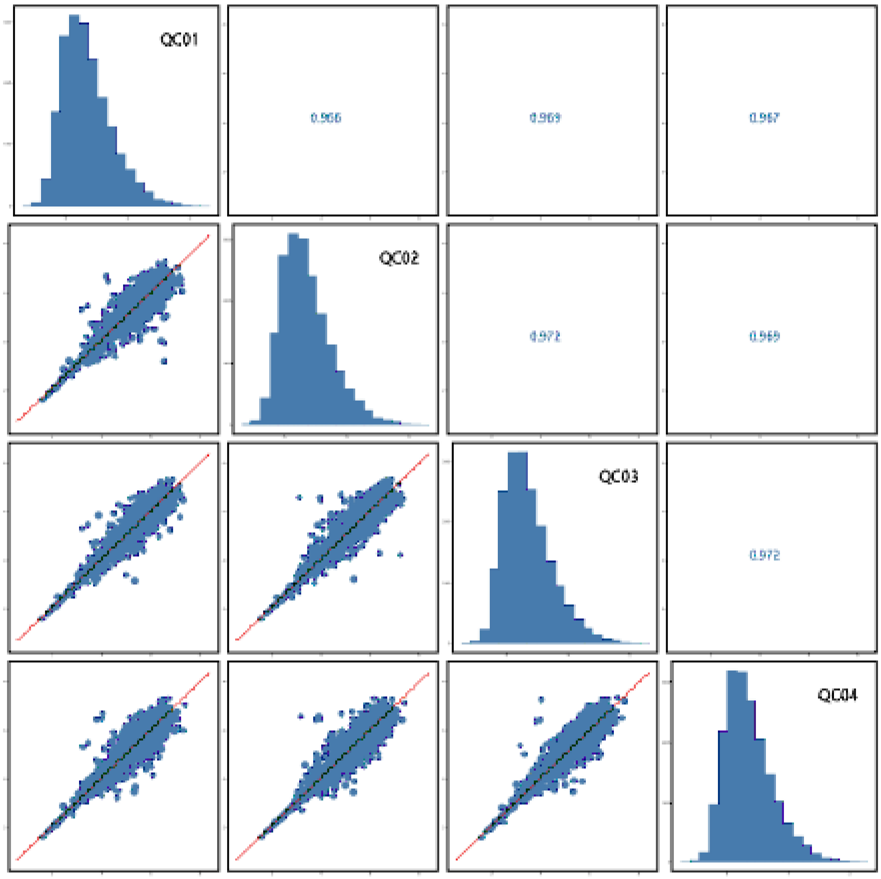

Figure S9. Correlation analysis of QC samples

Internal standards are introduced isotope-labeled metabolites. Since the internal standard concentrations in QC samples are the same, the smaller the response difference of internal standards (with a median RSD ≤ 10%), the more stable the system and the higher the data quality. It can be seen from the data in Table S1 that the data quality of this experiment is very high.

Table S1 Stability of internal standard responses in QC samples.

	rt	mz	rsd
IS1	116.6325	183.0824	0.0098
IS2	233.7685	152.0599	0.0508
IS3	166.642	133.1062	0.012
IS4	202.567	85.1322	0.0174
IS5	224.895	110.1083	0.0339
IS6	30.4754	127.0804	0.013
